# Physiological relevance and performance of a minimal lung model – an experimental study in healthy and acute respiratory distress syndrome model piglets

**DOI:** 10.1186/1471-2466-12-59

**Published:** 2012-09-21

**Authors:** Yeong Shiong Chiew, J Geoffrey Chase, Bernard Lambermont, Nathalie Janssen, Christoph Schranz, Knut Moeller, Geoffrey M Shaw, Thomas Desaive

**Affiliations:** 1 Department of Mechanical Engineering, University of Canterbury, Christchurch, New Zealand; 2 Medical Intensive Care Unit, University Hospital of Liege, Liege, Belgium; 3 Emergency Department, University Hospital of Liege, Liege, Belgium; 4 Institute for Technical Medicine, Furtwangen University, Villingen-Schwenningen, Germany; 5 Department of Intensive Care, Christchurch Hospital, Christchurch, New Zealand; 6 Thermodynamics of Irreversible Processes, Institute of Physics, University of Liege, Liege, Belgium

**Keywords:** ARDS, Recruitment model, Animal trials, Mechanical ventilation

## Abstract

**Background:**

Mechanical ventilation (MV) is the primary form of support for acute respiratory distress syndrome (ARDS) patients. However, intra- and inter- patient-variability reduce the efficacy of general protocols. Model-based approaches to guide MV can be patient-specific. A physiological relevant minimal model and its patient-specific performance are tested to see if it meets this objective above.

**Methods:**

Healthy anesthetized piglets weighing 24.0 *kg* [IQR: 21.0-29.6] underwent a step-wise PEEP increase manoeuvre from 5*cmH*_*2*_*O* to 20*cmH*_*2*_*O*. They were ventilated under volume control using Engström Care Station (Datex, General Electric, Finland), with pressure, flow and volume profiles recorded. ARDS was then induced using oleic acid. The data were analyzed with a Minimal Model that identifies patient-specific mean threshold opening and closing pressure (TOP and TCP), and standard deviation (SD) of these TOP and TCP distributions. The trial and use of data were approved by the Ethics Committee of the Medical Faculty of the University of Liege, Belgium.

**Results and discussions:**

3 of the 9 healthy piglets developed ARDS, and these data sets were included in this study. Model fitting error during inflation and deflation, in healthy or ARDS state is less than 5.0% across all subjects, indicating that the model captures the fundamental lung mechanics during PEEP increase. Mean TOP was 42.4*cmH*_*2*_*O* [IQR: 38.2-44.6] at PEEP = 5*cmH*_*2*_*O* and decreased with PEEP to 25.0*cmH*_*2*_*O* [IQR: 21.5-27.1] at PEEP = 20*cmH*_*2*_*O*. In contrast, TCP sees a reverse trend, increasing from 10.2*cmH*_*2*_*O* [IQR: 9.0-10.4] to 19.5*cmH*_*2*_*O* [IQR: 19.0-19.7]. Mean TOP increased from average 21.2-37.4*cmH*_*2*_*O* to 30.4-55.2*cmH*_*2*_*O* between healthy and ARDS subjects, reflecting the higher pressure required to recruit collapsed alveoli. Mean TCP was effectively unchanged.

**Conclusion:**

The minimal model is capable of capturing physiologically relevant TOP, TCP and SD of both healthy and ARDS lungs. The model is able to track disease progression and the response to treatment.

## Background

Mechanical ventilation (MV) is extensively used in the intensive care unit (ICU), to support and assist patients diagnosed with acute respiratory distress syndrome (ARDS). These patients have impaired lung function, and are extremely heterogeneous with significant inter- and intra- patient variation. Thus, patient-specific treatments are required to optimize outcome. Computer modeling can be used to identify and characterize patient-specific condition and guide clinical decisions [1-3]. Thus, the model’s physiological relevance corresponding to the patient disease state is crucial for its applicability in clinical decision support.

ARDS was first defined by Ashbaugh et al [4], as a consequence of variety of illness. They are characterized by fluid filled lungs (oedema), surfactant denature, causing alveolar instability and collapse, resulting in reduced in lung compliance and gas exchange [5]. A model that characterized the ARDS lung was proposed by Hickling [6]. It describes the lung as a collection of healthy and injured alveoli, distributed in layers subjected to a superimposed pressure. Healthy alveoli are normally open and assume a certain volume. Injured alveoli are collapsed and have no residual volume. They can be opened (recruited) with positive pressure through mechanical ventilation. Once opened, they will assume a volume similar to healthy alveoli. The opening and closing of collapsed alveoli are assumed to be governed by a normally distributed effective threshold opening pressure (TOP) and threshold closing pressure (TCP) [7,8]. Estimating the distribution of these parameters provides unique insight to patient-specific physiological condition, response to different MV treatment, and the opportunity to optimize patient-specific MV settings [9].

A healthy, spontaneously breathing lung normally has no collapsed alveoli. Thus, recruitment models are only considered applicable to characterize lung mechanics in ARDS or similar, which limits its application. A minimal model was proposed by Sundaresan et al using a similar, but modified recruitment concept and it was shown to be capable of monitoring the patient-disease state, predicting recruitment for changes in PEEP, and to guide MV therapy in the ICU [9,10]. It was able to identify physiologically relevant parameters that characterized patient-specific condition. However, the model is only used and tested in ARDS patients, and has yet to be validated for healthy lungs.

In this study, an animal trial is carried out to test the model’s physiological relevance and performance in both healthy and ARDS lungs. We hypothesize that the minimal model is able to represent both diseased and healthy lungs, as well as being able to monitor the progression of the disease state from the healthy case in a physiologically and clinically expected fashion. More specifically, it is assumed that the open alveoli in a healthy lung will have lower overall threshold opening pressures (lower mean TOP) compared to ARDS lungs, and that difference between healthy and ARDS states will be evident in lowered compliance and greater variability in threshold opening pressures (Higher standard deviation, SD). Satisfying these hypotheses would assist in validating the model’s application in MV patient.

## Methods

### Subject preparation

Experimental piglets were premedicated with tiletamin zolazepam 5 mg/kg and subsequently anaesthetized by a continuous infusion of sufentanil 0.5 μg/kg/h, pentobarbital 5 mg/kg/h and cisatracurium 2 mg/kg/h. They were ventilated through a tracheotomy under volume control (Tidal volume, Vt = 12 *ml/kg*) with inspired oxygen fraction (FiO_2_) of 0.5 using Engström Care Station (Datex, General Electric, Finland).

### Protocol-based recruitment manoeuvre

**Figure 1:**
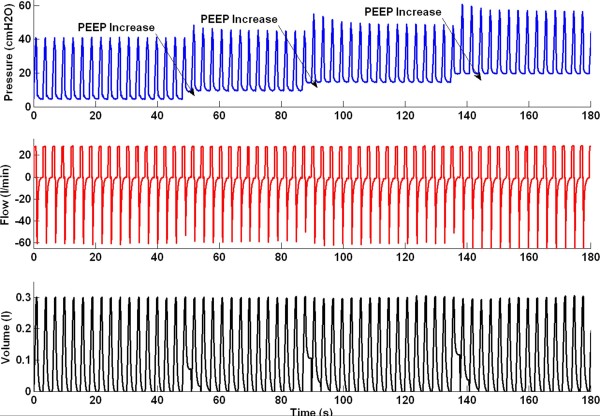
Pressure, flow and volume profile during recruitment manoeuvre.

Each subject underwent a protocol-based step-wise PEEP (positive end-expiratory pressure) increase recruitment manoeuvre (RM). Subjects were initially ventilated at baseline PEEP of 5*cmH*_*2*_*O*. During the RM, PEEP was increased with a 5c*mH*_*2*_*O* step until 20*cmH*_*2*_*O*. Other ventilator settings were maintained throughout the RM. Each PEEP level was maintained for 10 ~ 15 breaths before increasing to a higher PEEP level. Figure 1 shows an example of the continuously recorded airway pressure and flow during the RM.

After the RM, PEEP was decreased step-wise to baseline PEEP at 5*cmH*_*2*_*O*. At this PEEP, the healthy pigs were then injected with oleic acid to induce ARDS. Oleic acid was administrated slowly at 0.1 *ml* for every 10 minutes interval until 0.1 *ml/kg* of the subject’s weight. Arterial blood gases were monitored hourly, and, once diagnosed with ARDS, the subject underwent a second RM. In this study, ARDS criteria is limited to hypoxemia (PF ratio <200 *mmHg*). All experimental procedure, protocols and the use of data in this study were reviewed and approved by the Ethics Committee of the University of Liege Medical Faculty.

### Data processing

**Figure 2:**
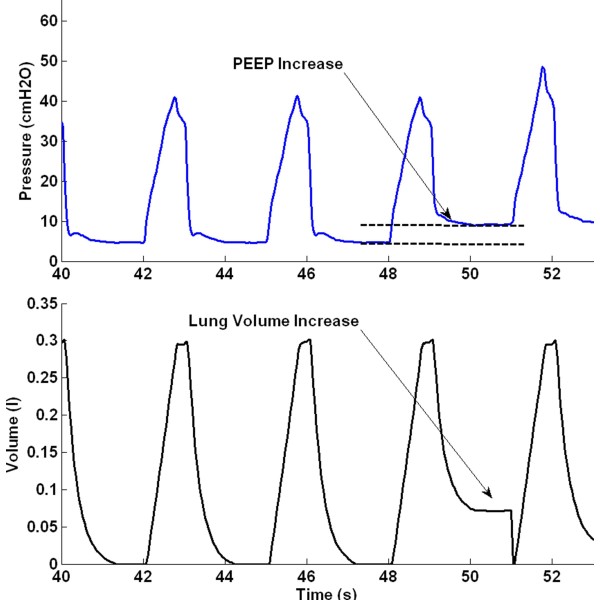
Estimation of volume increase during PEEP increment.

**Figure 3:**
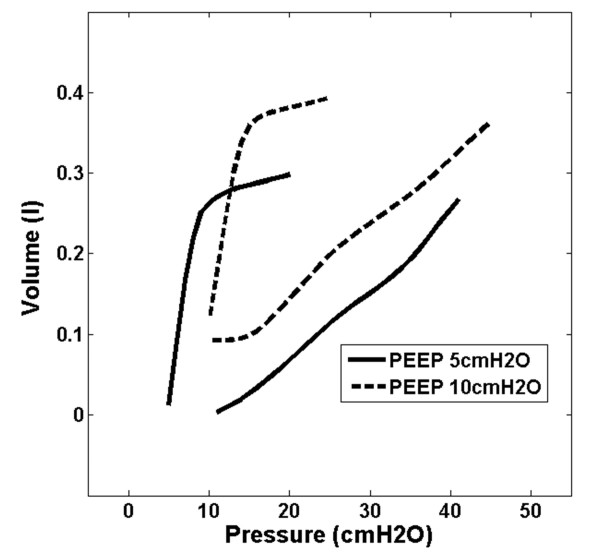
Example of pressure volume curves with volume increase with PEEP.

A representative breath is selected from the last 2 breaths at each PEEP level, with the assumption of viscoelastic stabilization has occurred after PEEP increase. When PEEP increases from a lower to higher level, recruitment occurs and the deflation/unloading of the lung is not complete, with additional air “trapped” in lung. This recruitment or “trapped” volume is the estimated lung volume increase for the PEEP increment. Figure 2 shows an example of the estimated lung volume increase, and the associated post-processed pressure volume curve (PV) is shown in Figure 3.

### Model fitting and data analysis

The PV curves were fitted to a minimal model [10] to identify model-based mean TOP and TCP, and the standard deviation (SD) of the TOP and TCP distributions. The minimal model is based on the concept of recruitment, and assumes the lung is a collection of lung units that are either open or collapsed. During inflation, if airway pressure exceeds a lung unit’s effective TOP, the lung unit will assume a lung unit volume. Each opened unit volume is added to form the inflation PV curve. Similarly, if the airway pressure during deflation drops below the effective closing pressure, the lung unit collapses and loses the unit volume, which forms the deflation curve. Each lung unit has different effective opening pressure and closing pressure, and they are assumed normally distributed, so only a mean and SD of the distribution needs to be estimated [7,8,11]. The model summary is shown in Equation 1 and the details of the model can be found in [10]

(1) $$\text{Volume}\;\left(\text{Pressure}\right)=\frac{1}{2}\left[1+erf\left(\frac{\mathrm{Pr}essure-Mean}{\sqrt{2\times SD^{2}}}\right)\right]*Total\;Lung\;Capacity$$

Where *erf* is the Gaussian Error Function.

In a TOP distribution, the mean of the distribution is the pressure when the maximum rate of recruitment occurs. The mean TOP also indicates the mean recruitable total lung units when ventilated at that pressure. Equally, the mean of TCP distribution indicates the maximum rate of derecruitment during deflation and, the mean lung units that will remain recruited during deflation. The SD describes the shape of the TOP/TCP distribution and is an indication of lung heterogeneity. SD reflects compliance and varies for a given subject, depending on the lung condition.

**Figure 4:**
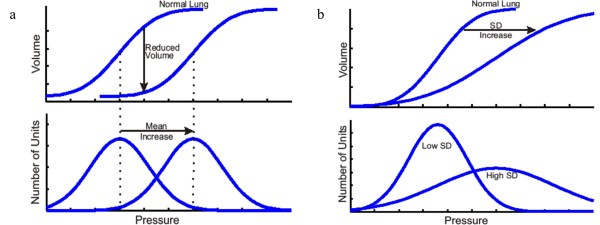
**Effect of TOP and SD towards a PV curve.** (**a**) Normal lung to collapse Lung. (**b**) Normal lung to heterogeneous lung (Top – PV curve during inflation, Bottom – TOP distribution based on PV curves).

Figure 4 shows examples of how different lung conditions affect the TOP distribution. The upper figures are the inspiratory PV curves and the lower figures the corresponding TOP distribution. A collapsed lung requires higher pressure to open/recruit the lung units, therefore, mean TOP thus increases as shown in Figure 4(a). The SD is the “spread” of the TOP distribution and thus, a heterogeneous lung will result in higher SD, as shown in Figure 4(b). Combination of TOP and SD will thus give the information of overall lung compliance. Similar concepts apply to the TCP distribution.

In this study, the PV curves were fitted to clinical data using this minimal model [10]. Fitting errors are presented as mean absolute percentage error to indicate model performance. Wilcoxon rank-sum test is used to test for any statistical significance. Model-based mean TOP, TCP and SD in both healthy and ARDS states are compared to examine the effect of ARDS on model parameters, and their physiological and clinical relevance.

### Disease state grouping (DSG)

**Figure 5:**
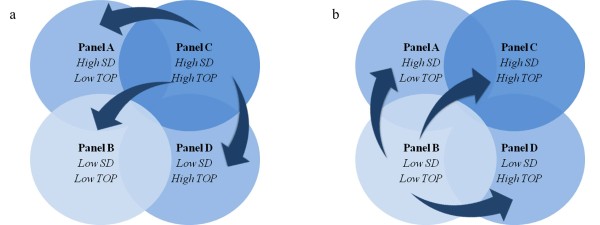
**Patients-specific disease state grouping and tracking.** (**a**) Lung is recovering over time. (**b**) Lung condition worsening.

The estimated patient-specific parameters (mean TOP and SD) can be used to group patients based on their disease state using the 4 panel disease state grouping metric (DSG) shown in Figure 5(a) and 5(b). In general, patients grouped in Panel B (low SD and TOP) are healthier compared to other panels. A decrease of SD or mean TOP indicate a less heterogeneous lung and/or an overall decrease in collapsed lung units. Figure 5(a) showed improvement in lung condition. Conversely, an increase of either of these parameters indicates that lung condition is worsening over time as shown in Figure 5(b). Hard boundaries are deliberately not shown as specific because it may be patient- or group-specific, and hard to define without debate with data available today.

## Results

9 piglets weighing median [Interquartile range (IQR)] 24.0 *kg* [IQR: 21.0-29.6] were included in the study. 3 of 9 subjects reached an ARDS state (Subjects 5, 6 and 9) after oleic acid injection. Individual model parameters are compared between the healthy and ARDS state for these 3 piglets. The summary of model fitting during inflation or deflation for healthy and ARDS subjects is shown in Table 1. The details of Table 1 can be found in the Additional file 1: Table E1-E3.

**Table 1:** Model fitting error (median [IQR]) during inflation and deflation at different PEEP levels for healthy and ARDS subjects

**Inflation**	**Absolute percentage fitting error (%)**				**Median [IQR]**
	**PEEP 5**	**PEEP 10**	**PEEP 15**	**PEEP 20**	
**Healthy State Inflation**	6.59 [4.87-8.45]	3.59 [2.67-5.15]	2.55 [2.16-2.93]	0.78 [0.43-0.99]	3.06 [2.62-3.70]
**Healthy State Deflation**	9.86 [6.23-11.44]	2.51 [1.84-6.49]	1.37 [1.01-3.47]	0.98 [0.61-1.87]	1.78 [1.56-4.98]
ARDS State* Inflation	10.68	6.25	2.94	1.57	4.60 [2.26-8.47]
ARDS State* Deflation	7.72	2.80	1.66	1.20	2.23 [1.43-5.26]

*Fitting errors for ARDS state at PEEP 5, 10, 15 and 20*cmH2O* are average values instead of median [IQR].

Table 2 shows the model estimated mean TOP and TCP at different PEEP for healthy subjects, and Table 3 for the ARDS subjects (5, 6 and 9). Table 4 shows the SD of the TOP and TCP distribution for the subjects which developed ARDS in both healthy and ARDS state.

**Table 2:** Mean TOP and TCP for healthy subjects

**Subject**	**Threshold opening pressure**				**Threshold closing pressure**			
	**(TOP,*cmH***_***2***_***O*)**				**(TCP,*cmH***_***2***_***O*)**			
	**PEEP 5**	**PEEP 10**	**PEEP 15**	**PEEP 20**	**PEEP 5**	**PEEP 10**	**PEEP 15**	**PEEP 20**
**1**	42.4	39.2	32.9	27.2	9.6	13.4	16.6	19.8
**2**	36.3	40.0	33.8	29.3	8.2	13.5	16.8	20.4
**3**	44.6	37.4	32.2	25.1	10.2	13.2	16.4	19.7
**4**	39.0	33.9	29.0	19.2	10.4	13.7	16.6	19.0
**5**	42.6	32.7	24.8	19.2	10.2	13.2	15.7	18.4
**6**	31.3	28.1	23.8	21.5	9.03	12.5	15.5	18.8
**7**	47.7	40.8	34.1	27.0	11.1	14.6	17.2	19.6
**8**	46.0	40.2	33.4	27.9	10.6	13.4	16.6	19.5
**9**	38.2	33.9	29.4	22.8	8.7	12.3	15.8	19.3
**Median [IQR]**	42.4 [38.2-44.6]	37.4 [33.9-40.0]	32.2 [29.0-33.3]	25.0 [21.5-27.1]	10.2 [9.0-10.4]	13.3 [13.2-13.5]	16.6 [15.8-16.6]	19.5 [19.0-19.7]

**Table 3:** Mean TOP and TCP for ARDS subjects

**Subject**	**Threshold Opening Pressure**				**Threshold Closing Pressure**			
	**(TOP,*cmH***_***2***_***O*)**				**(TCP,*cmH***_***2***_***O*)**			
	**PEEP 5**	**PEEP 10**	**PEEP 15**	**PEEP 20**	**PEEP 5**	**PEEP 10**	**PEEP 15**	**PEEP 20**
**5**	48.1	44.1	33.3	22.7	10.2	14.0	16.7	19.0
**6**	49.5	41.6	31.1	19.1	10.2	13.7	16.7	18.9
**9**	68.1	64.7	58.7	49.4	9.6	14.1	18.2	21.8
**Average**	55.2	50.1	41.0	30.4	10.0	13.9	17.2	19.9

**Table 4:** SD in healthy and ARDS lung

**Subject**	**Healthy**		**ARDS**	
	**Inflation**	**Deflation**	**Inflation**	**Deflation**
**5**	23	4	25	4
**6**	14	3	25	4
**9**	21	3	23	3
**Average**	19.3	3.3	24.3	3.7

**Figure 6:**
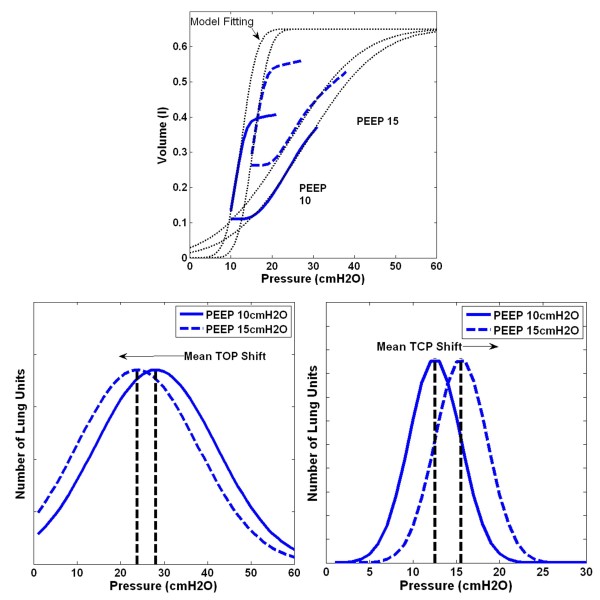
**Model Fitting with TOP and TCP distribution shift for healthy Subject 2.** (Top - Model Fitting for PV curve in PEEP 10 and 15*cmH*_*2*_*O*, Bottom - TOP shifts left and TCP shifts right with PEEP increase.

**Figure 7:**
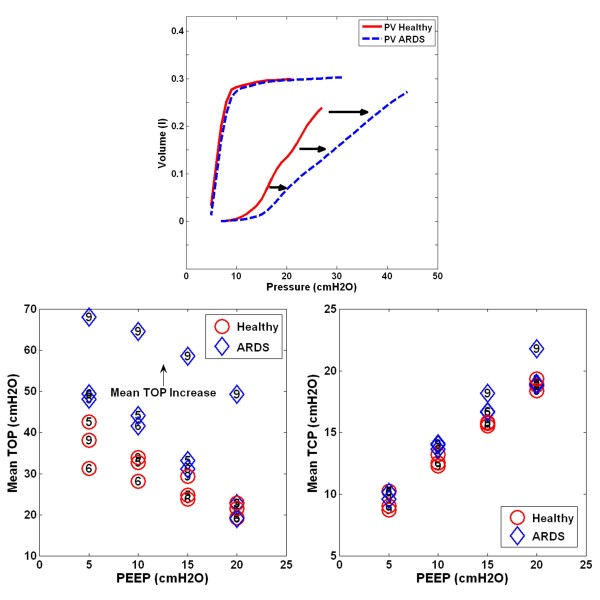
**Pressure-volume curve of Subject 5 and overall TOP and TCP comparison between healthy and ARDS.** (Top - Inflation curve right shift from healthy to ARDS, Bottom - TOP in healthy lung is lower than in ARDS. Relatively little change in TCP during healthy and ARDS state).

**Figure 8:**
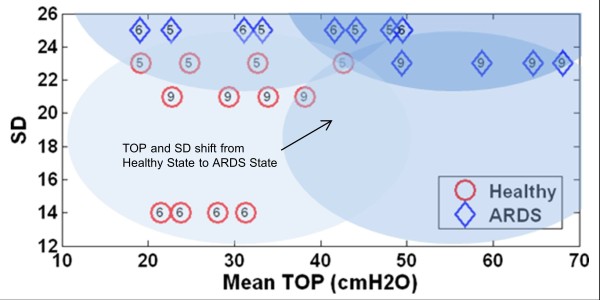
**Mean TOP and SD change for healthy subject which later develop ARDS.** (**a**) Subject 5, with slight increase of SD and TOP. (**b**) Subject 6, large increase of SD. (**c**) Subject 9, slight increase of SD with high TOP change.

Figure 6 shows the model fit to measured PV curves of a healthy subject, and the resulting TOP and TCP distributions at PEEP of 10*cmH*_*2*_*O* and 15*cmH*_*2*_*O*. An example of the PV curve shift from a healthy state to an ARDS state is shown in Figure 7 (Upper). The change in TOP and TCP distributions between healthy and ARDS state for the 3 subjects in Tables 2-3 is shown in Figure 7 (bottom). Figure 8 shows these changes in the disease state grouping (DSG) for Subjects 5, 6 and 9.

## Discussion

The median fitting errors in healthy subjects during inflation and deflation were less than 3.1%. Similar to healthy subjects, the model fits well for ARDS subjects with median absolute percentage error during inflation and deflation less than 4.7%. There is a noticeable high median fitting error for ARDS subject 9 at PEEP 5*cmH*_*2*_*O*, at 27.32% during inflation. The model was not able to capture these specific physiological conditions at low PEEP. In particular, this case can be associated with the effect of Auto-PEEP distorting the actual lung condition [9]. The recruitment model fits better when Subject 9 is ventilated at higher PEEP (P < 0.005) compared to lower PEEP. However, the relatively low median error overall subjects indicates the model is capable of capturing fundamental mechanics of both healthy and ARDS lungs.

Tables 2-3 show the estimated mean TOP and TCP for all the healthy and ARDS subjects. In healthy subjects, the overall mean TOP is decreased with increasing PEEP. Mean TCP increases with increasing PEEP. The TOP and TCP distribution shift of a subject during PEEP increase is observed in Figure 6 (Bottom), and are capturing the recruitment as expected.

Similar mean TOP and TCP trends are also observed in ARDS subjects. However, an overall higher TOP is observed compared to healthy subjects, which is also expected for an ARDS lung. The overall higher mean TOP indicates that the ARDS lung consists of relatively more collapsed alveoli and higher pressure is needed to recruit the collapsed alveoli.

Healthy lungs normally consist of only opened or recruited lung units, and a model based on the concept of recruitment may not be applicable. However, in a healthy anesthetized and sedated subject, pulmonary atelectasis can be observed, but it is less severe compared to an ARDS lung and can be easily recruited [12-14]. Thus, during inflation, relatively lower pressure is needed to ventilate the healthy “collapsed” lung compared to ARDS lung. Therefore, for a given tidal volume, the area within the PV curve for a healthy lung should be smaller than ARDS lung. Equally, the healthy lung is less heterogeneous and the lower SD will keep the PV loop area smaller. Figure 7 shows a clear comparison of a healthy and ARDS PV curve, in which the ARDS PV curve has greater area than the healthy PV curve and correspondingly higher SD for this Subject 5 in Table 4. The change thus shows the expected higher work of breathing in the heterogeneous ARDS lung.

Comparing the healthy and ARDS state, mean TOP for healthy lungs are lower when compared to ARDS lungs in Figure 7 (Bottom Left). A healthy lung is a less heterogeneous lung and the effect of superimposed pressure to alveoli is less detrimental. As suggested earlier, a healthy lung is normally open, which results in a lower mean TOP. Thus, the model captures the fact that, for the same subject at a healthy and ARDS state, higher pressure is required to recruit and open the lung. The inter-subject variability in this behavior is evident in Figure 8. Overall, these model results match clinical observation and expectation, which further validates the model.

The deflation curve remains unchanged in ARDS compared to healthy subjects, as shown in Figure 7 (Top), which results in relatively no change in TCP, as seen in Figure 7 (Bottom Right) and Table 3. Hypothetically, mean TCP should be higher in the ARDS state compared to the healthy state [10,11]. ARDS lung units are more unstable and vulnerable to collapse. Thus, higher pressure is required to retain recruitment. However, this hypothesis was neither observed nor apparent in these results. Only a small increase in TCP is observed during ARDS state compared to healthy state as shown in Figure 7.

The DSG for the ARDS subjects are shown in Figure 8. It is observed that all 3 subjects experienced different SD and TOP increase when transitioning from healthy to ARDS state. In particular, Subject 5 has a relatively small increase in both SD and TOP between healthy and ARDS state. Subject 6 had very large increase in SD (heterogeneity) but less change in TOP (Collapsed lung units). Subject 9 had a very high TOP change (Lung collapse) but minimal changes in SD (Heterogeneity). These results show the diversity in the impact of the ARDS induced.

It is known that ARDS induced in animal model using oleic acid are highly variable [15]. A small variation in ventilation and hemodynamic management during preparation, time and dosage may alter the severity or extensiveness of the lung injury, resulting in different pathophysiological consequences [15-18]. That behavior is clearly evident in these results.

Importantly, this research focuses on minimal model performance in healthy and ARDS lungs. Combining the DSG for all 3 subjects, as shown in Figure 8, the healthy subjects have overall lower TOP and SD than in the ARDS state. This finding suggests that the DSG application is not limited to patient-specific disease state tracking, and it is possible to be expanded into population monitoring. Capturing 3 different ARDS respiratory mechanics or pathophysiological consequences, thus encourages the model’s application in clinical setting, where the presentation of ARDS and its evolution over time and treatment can be variable.

This DSG application is unique and observing DSG shifts should provide useful information for clinical decision support. For example, patients who are grouped in Panel D (High TOP, low SD), have a less heterogeneous lung, but with overall higher lung unit opening pressure. For example, it is hypothesized that a high PEEP can be used in MV to recruit overall collapsed lung units and improve gas exchange [19,20]. For patients who are grouped in Panel A (Low TOP, high SD), ventilation modes with 2 PEEP levels (Bi-Level PEEP ventilation, airway pressure release ventilation (APRV)) can reduce cyclic opening and collapse of lung units and improve patient outcome [21-23]. Tracking patient DSG with time will also show the effect and patient’s response to specific treatment. In this research, the effect of oleic acid can be seen in increase of TOP and SD. However, the exact limits of these groupings remain to be determined, although it does not affect the ability to track patient condition and response to therapy as in Figure 8.

Overall, the difference of mean TOP and SD between the healthy and ARDS state can be identified using the minimal model. The application of minimal model is not limited to the diseased lung, and allows comparison between healthy and ARDS lungs, and thus encourages its application and future investigation in the ICU to monitor patients-specific condition to guide MV therapy. An overall down shift of mean TOP and/or lowered SD will indicate that the lung recovering for injurious state. In contrast, an up-shift of TOP and/or SD, will show that the lung is more injured. This unique pair of metric thus provides the ability to track the disease state from healthy to injured state and vice-versa. However, mean TCP appears to have little change between healthy and ARDS state, indicating that the TCP parameter was less significant in this clinical use.

## Limitations

### ARDS piglets

After oleic acid injections, only 3 of 9 subjects successfully developed ARDS. Others experienced hemodynamic failure before ARDS could develop fully or detected. This result shows that oleic acid induced ARDS animals are less reproducible and the subject preparation method should be re-examined [15,24-26]. The estimation and comparison of TOP, TCP and SD during healthy and ARDS state is thus, not conclusive with statistical significance given low subject numbers. However, individual data revealed that subjects that developed ARDS had overall higher TOP compared to subject in a healthy state. This physiologically relevant result is supported by past literature that examines similar clinical conditions [7,8,27]. In addition, all other results follow clinically expected trends.

### Ventilation tidal volume

In this study, tidal volume is set to 12 *ml/kg* to ventilate the experimental piglets. It is known that such a high tidal volume is injurious with higher mortality [28]. However, the focus of the study is the investigation of the model’s performance in healthy and ARDS states. During a healthy state, the recruitment manoeuvre with airway pressure and flow measurements were performed at the very beginning of the trial. This time frame is relatively short and thus, the effect of high tidal volume ventilation was minimal and likely did minimal or no damage. Moreover, a more injurious ventilation strategy would indirectly benefit the overall study goals comparing healthy and damaged lung state.

### Estimation of the volume change

The measurement of volume change was estimated during RM PEEP increase. The calculation method assumes that deflation of the lung is not fully complete and the air remained in the lung due to PEEP. This estimation based on Figure 2 may not be entirely true. However, direct measurement of the lung volume during short PEEP increases is not available at the bedside. In particular, FRC estimation using nitrogen washout requires several breathing cycles and a long stabilization period and thus, was not suitable in this trial or for regular clinical use (1-4 times per day). The volume change estimation is this study is thus a surrogate of the actual lung volume increase. This estimation method can be validated in future studies using nitrogen washin/washout method. However, all trends remain valid, and it is these changes that are critical here. Equally, low fitting errors indicate it did not appear to affect the model.

### Minimal model and patient DSG

The minimal model is a model that estimates TOP, TCP and SD during PEEP titration of the mechanically ventilated. It is unable to predict the alveolar over-distension directly. However, the use of TOP mean shift as proposed by Sundaresan et al [9], it is possible to monitor the recruitability of the lung and thus, indirectly reveal potential over-distension that may cause lung injury.

The DSG provides a unique metric to monitor patient’s condition and potentially be used to guide ventilator settings. However, there are currently insufficient samples to validate this metric, or to prove the patients outcome for different TOP and SD. In particular, questions such as: “what is the actual physiological findings in patients with particular SD/TOP value”, “what SD or TOP value are considered as high or low” need to be addressed. Figure 8 is an example of the metric application, but there is insufficient information to determine which specific TOP/SD is high/low. In addition, the estimated TOP and SD in animals may be different if compared with human subjects. Future clinical trials or clinical PV data from other trials are required to validate this proof of concept.

## Conclusions

The minimal model fits well in both healthy and ARDS lungs, and is capable of capturing the fundamental lung mechanics of the healthy and ARDS lung. The application of minimal model is thus not limited to diseased lung cases, but can be even used for healthy lungs. The model was able to estimate clinically and physiological relevant parameters for healthy and ARDS piglets thus allowing disease state tracking (DSG), which in turn reveals a potential to use this model to assist in clinical decision making.

## Abbreviations

APRV: Airway release pressure ventilation; ARDS: Acute respiratory distress syndrome; DSG: Disease state grouping; FiO_2_: Inspired oxygen fraction; ICU: Intensive care unit; IQR: Interquartile range; MV: Mechanical ventilation; PEEP: Positive end expiratory pressure; PV: Pressure volume; RM: Recruitment manoeuvre; SD: Standard deviation; TCP: Threshold closing pressure; TOP: Threshold opening pressure; Vt: Tidal volume.

## Competing interest

The authors declare that they have no conflict of interest.

## Authors’ contributions

YSC, GMS and JGC created and defined the model. BL, NJ and TD implemented trials clinically with input from all others. Every author had input in analysis of results, writing and revising the manuscript. All authors read and approved the final manuscript.

## Pre-publication history

The pre-publication history for this paper can be accessed here:

http://www.biomedcentral.com/1471-2466/12/59/prepub

## Supplementary Material

Additional file 1 **Table E1, E2 and E3.** shows the detail information on model fitting error during inflation, deflation, healthy and ARDS state at different PEEP. Results are presented in median and interquartile range [IQR]. **Table E4** shows the peak airway pressure for every subject at different PEEP. **Table E5** shows the static compliance for every subject at different PEEP. (DOCX 30 kb) Click here for file
