# Supplementary material for: Physiological relevance and performance of a minimal lung model – an experimental study in healthy and acute respiratory distress syndrome model piglets
Source: BMC Pulm Med. 2012 Sep 21;12:59. doi: 10.1186/1471-2466-12-59 (PMC3511291; doi:10.1186/1471-2466-12-59)
Supplement: Additional file 1 — Table E1, E2 and E3. shows the detail information on model fitting error during inflation, deflation, healthy and ARDS state at different PEEP. Results are presented in median and interquartile range [IQR]. Table E4 shows the peak airway pressure for every subject at different PEEP. Table E5 shows the static compliance for every subject at different PEEP. (DOCX 30 kb) [file 1471-2466-12-59-S1.docx]

**Electronic Supplemental file**

Table E1, E2 and E3 shows the detail information on model fitting error during inflation, deflation, healthy and ARDS state at different PEEP. Results are presented in median and interquartile range [IQR].

Table E4 shows the peak airway pressure for every subject at different PEEP.

Table E5 shows the static compliance for every subject at different PEEP.

**Table E1:** Model fitting error (median [IQR]) during Inflation at different PEEP levels for healthy subjects

| **Subject** | **Absolute Percentage Fitting Error (%), Inflation** | | | | **Median of Medians [IQR]** |
| --- | --- | --- | --- | --- | --- |
|  | **PEEP 5** | **PEEP 10** | **PEEP 15** | **PEEP 20** |  |
| **1** | 3.87  [2.38-5.07] | 6.76  [3.91-8.06] | 2.92  [1.69-3.40] | 0.87  [0.57-1.09] | 3.40  [1.90-5.32] |
| **2** | 5.50  [3.25-8.17] | 5.02  [2.31-6.90] | 2.21  [0.89-2.97] | 0.31  [0.16-0.43] | 3.61  [1.26-5.26] |
| **3** | 6.59  [4.63-26.15] | 4.79  [2.59-6.10] | 3.35  [1.69-4.62] | 0.91  [0.51-1.24] | 4.07  [2.13-5.69] |
| **4** | 4.52  [3.96-10.07] | 3.58  [1.66-4.63] | 2.55  [1.40-3.35] | 0.44  [0.13-0.84] | 3.06  [1.49-4.05] |
| **5** | 11.83  [4.43-25.55] | 1.12  [0.37-1.55] | 1.28  [0.78-2.33] | 0.78  [0.44-0.98] | 1.20  [0.95-6.56] |
| **6** | 10.01  [7.19-36.81] | 1.84  [0.90-2.87] | 2.48  [1.66-2.67] | 0.73  [0.52-2.61] | 2.16  [1.29-6.25] |
| **7** | 6.59  [5.15-19.09] | 2.95  [1.65-3.80] | 2.91  [1.91-3.53] | 1.21  [0.70-1.56] | 2.93  [2.06-4.77] |
| **8** | 4.98  [2.87-6.82] | 5.55  [3.14-7.51] | 2.93  [1.65-3.65] | 0.40  [0.21-0.50] | 3.96  [1.67-5.27] |
| **9** | 7.92  [6.77-33.45] | 3.39  [2.13-7.44] | 2.02  [1.68-2.26] | 2.15  [1.68-2.85] | 2.77  [2.09-5.66] |
| **Median of Medians [IQR]** | 6.59  [4.87-8.45] | 3.59  [2.67-5.15] | 2.55  [2.16-2.93] | 0.78  [0.43-0.99] | 3.06  [2.62-3.70] |

**Table E2:** Model fitting error during Deflation at different PEEP levels for healthy subjects

| **Subject** | **Absolute Percentage Fitting Error (%), Deflation** | | | | **Median of Medians [IQR]** |
| --- | --- | --- | --- | --- | --- |
|  | **PEEP 5** | **PEEP 10** | **PEEP 15** | **PEEP 20** |  |
| **1** | 14.24  [6.54-21.03] | 4.38  [2.07-6.07] | 3.18  [2.03-3.66] | 1.74  [1.08-1.89]] | 3.78  [2.46-9.31] |
| **2** | 11.68  [5.26-21.86] | 6.49  [2.17-13.04] | 3.29  [1.90-4.50] | 0.98  [0.53-1.47] | 4.89  [2.14-9.08] |
| **3** | 5.66  [4.56-15.35] | 2.47  [0.95-3.86] | 0.91  [0.81-1.02] | 1.08  [0.59-1.37] | 1.78  [0.99-4.07 |
| **4** | 9.93  [3.75-16.1] | 1.87  [1.39-2.12] | 1.16  [0.56-1.41] | 0.09  [0.06-0.29] | 1.51  [0.63-5.90] |
| **5** | 8.94  [1.35-16.52] | 1.77  [0.83-2.71] | 1.37  [1.11-2.85] | 0.80  [0.49-0.83] | 1.57  [1.08-5.35] |
| **6** | 4.53  [1.82-7.26] | 1.12  [0.94-1.30] | 1.01  [0.09-1.93] | 0.50  [0.15-1.36] | 1.07  [0.75-2.83] |
| **7** | 6.42  [3.88-17.22] | 2.51  [0.76-4.22] | 1.01  [0.48-1.15] | 0.65  [0.30-0.66] | 1.76  [0.83-4.47] |
| **8** | 9.86  [6.73-17.37] | 6.52  [3.09-7.83] | 4.03  [1.95-4.72] | 2.25  [1.12-2.97] | 5.27  [3.14-8.19] |
| **9** | 11.37  [11.29-13.02] | 10.67  [9.55-13.42] | 9.75  [9.00-11.08] | 4.78  [4.12-4.85] | 10.21  [7.26-11.02] |
| **Median of Medians [IQR]** | 9.86  [6.23-11.44] | 2.51  [1.84-6.49] | 1.37  [1.01-3.47] | 0.98  [0.61-1.87] | 1.78  [1.56-4.98] |

**Table E3:** Model fitting error for subjects with ARDS

| **Subject** | **Absolute Percentage Fitting Error (%)**  **Inflation** | | | | **Median of Medians [IQR]** |
| --- | --- | --- | --- | --- | --- |
|  | **PEEP 5** | **PEEP 10** | **PEEP 15** | **PEEP 20** |  |
| **5** | 2.75  [1.49-3.45] | 7.51  [3.11-11.45] | 2.92  [1.98-4.28] | 0.53  [0.31-1.59] | 2.84  [1.64-5.22] |
| **6** | 1.97  [0.70-3.15] | 5.34  [3.24-8.58] | 4.40  [2.34-5.28] | 0.62  [0.19-0.82] | 3.19  [1.30-4.87] |
| **9** | 27.32  [12.54-59.58] | 5.92  [2.08-10.06] | 1.48  [0.83-3.57] | 3.57  [2.55-4.31] | 4.74  [2.53-16.62] |
| **Average** | 10.68 | 6.25 | 2.94 | 1.57 | 4.60  [2.26-8.47] |
|  | **Deflation** | | | |  |
|  | **PEEP 5** | **PEEP 10** | **PEEP 15** | **PEEP 20** |  |
| **5** | 10.79  [5.71-14.25] | 3.89  [1.98-5.34] | 0.83  [0.38-1.41] | 1.73  [1.00-1.94] | 2.81  [1.28-7.34] |
| **6** | 6.16  [4.53-15.62] | 1.14  [0.89-1.49] | 3.45  [3.38-3.92] | 0.30  [0.13-0.62] | 2.30  [0.72-4.81] |
| **9** | 6.22  [2.90-9.78] | 3.37  [0.38-5.87] | 0.70  [0.54-2.95] | 1.55  [0.86-2.53] | 2.46  [1.13-4.79] |
| **Average** | 7.72 | 2.80 | 1.66 | 1.20 | 2.23  [1.43-5.26] |

**Table E4:** Peak pressure for every subject at different PEEP

| **Healthy State Subject** | **Peak Pressure (*cmH_2_O*)** | | | |
| --- | --- | --- | --- | --- |
|  | **PEEP 5** | **PEEP 10** | **PEEP 15** | **PEEP 20** |
| **1** | 42.0 | 49.5 | 51.2 | 63.0 |
| **2** | 36.4 | 42.3 | 48.9 | 60.8 |
| **3** | 37.4 | 42.3 | 48.5 | 57.0 |
| **4** | 31.5 | 37.3 | 44.0 | 52.8 |
| **5** | 27.7 | 32.7 | 40.6 | 58.4 |
| **6** | 26.9 | 31.9 | 38.9 | 57.5 |
| **7** | 35.7 | 38.8 | 43.3 | 50.5 |
| **8** | 42.2 | 47.3 | 51.2 | 59.6 |
| **9** | 34.8 | 38.3 | 42.2 | 52.9 |
| **Median**  **[IQR]** | 35.7  [30.6-38.6] | 38.8  [36.2-43.6] | 44.0  [41.8-49.5] | 57.5  [52.9-59.9] |
| **ARDS State Subject** | **Peak Pressure (*cmH_2_O*)** | | | |
|  | **PEEP 5** | **PEEP 10** | **PEEP 15** | **PEEP 20** |
| **5** | 44.6 | 51.4 | 52.1 | 63.2 |
| **6** | 41.3 | 45.1 | 48.7 | 56.1 |
| **9** | 62.7 | 63.9 | 65.4 | 68.2 |
| **Average** | 49.5 | 53.5 | 55.4 | 62.5 |

**Table E5:** Static Compliance for every subject at different PEEP.

| **Healthy State Subject** | **Static Compliance (*ml/cmH_2_O*)** | | | |
| --- | --- | --- | --- | --- |
|  | **PEEP 5** | **PEEP 10** | **PEEP 15** | **PEEP 20** |
| **1** | 9.62 | 9.42 | 10.51 | 8.07 |
| **2** | 11.90 | 11.36 | 10.90 | 8.52 |
| **3** | 11.98 | 12.23 | 11.45 | 9.73 |
| **4** | 12.77 | 12.12 | 11.31 | 9.61 |
| **5** | 16.10 | 15.88 | 14.07 | 8.76 |
| **6** | 17.14 | 16.69 | 14.98 | 9.26 |
| **7** | 13.25 | 14.11 | 14.14 | 12.83 |
| **8** | 10.25 | 10.08 | 10.68 | 8.96 |
| **9** | 13.94 | 14.44 | 15.07 | 12.05 |
| **Median**  **[IQR]** | 12.77  [11.49-14.48] | 12.23  [11.04-14.80] | 11.45  [10.85-14.35] | 9.26  [8.70-10.31] |
| **ARDS State Subject** | **Static Compliance (*ml/cmH_2_O*)** | | | |
|  | **PEEP 5** | **PEEP 10** | **PEEP 15** | **PEEP 20** |
| **5** | 9.17 | 8.63 | 9.86 | 8.23 |
| **6** | 10.58 | 10.87 | 10.51 | 10.16 |
| **9** | 7.35 | 8.05 | 8.62 | 8.78 |
| **Median**  **[IQR]** | 9.03 | 9.18 | 9.66 | 9.06 |
